# Bis(μ-*N*,*N*′,*N*′′-tri-3-pyridylpyridine-1,3,5-tricarboxamide-κ^2^ *N*:*N*′)bis­[di­chloridomercury(II)] methanol disolvate

**DOI:** 10.1107/S160053681102040X

**Published:** 2011-06-04

**Authors:** Pei Wang, Yufei Wang, Chao Huang, Lixiang Chang, Jie Wu

**Affiliations:** a Department of Chemistry, Zhengzhou University, Zhengzhou 450052, People’s Republic of China; b College of Chemical Engineering and Food Science, Zhongzhou University, Zhengzhou 450044, People’s Republic of China

## Abstract

The title dinuclear centrosymmetric complex, [Hg_2_Cl_4_(C_24_H_18_N_6_O_3_)_2_]·2CH_3_OH, comprises Hg^II^ atoms coordinated by two Cl atoms and two N atoms from ligands in a distorted tetra­hedral geometry. The solvent mol­ecules are linked by hydrogen bonds.

## Related literature

For general background, see: Fortner *et al.* (2005 ▶). For a related structure, see: Qin *et al.* (2003 ▶).
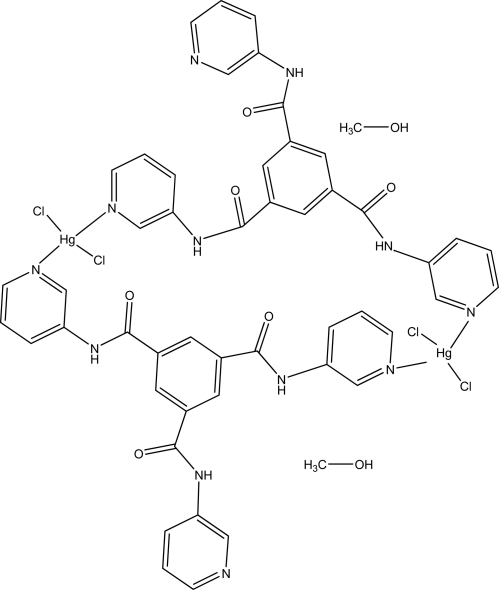


## Experimental

### 

#### Crystal data

[Hg_2_Cl_4_(C_24_H_18_N_6_O_3_)_2_]·2CH_4_O
*M* *_r_* = 1483.95
Triclinic, 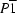

*a* = 8.6772 (17) Å
*b* = 12.243 (2) Å
*c* = 13.530 (3) Å
α = 66.81 (3)°
β = 84.66 (3)°
γ = 86.40 (3)°
*V* = 1315.0 (4) Å^3^
*Z* = 1
Mo *K*α radiation
μ = 6.10 mm^−1^
*T* = 293 K
0.20 × 0.18 × 0.16 mm

#### Data collection

Rigaku Saturn724 diffractometer
Absorption correction: numerical (*CrystalClear*; Rigaku/MSC, 2006 ▶) *T*_min_ = 0.738, *T*_max_ = 1.000
14489 measured reflections
5170 independent reflections
4461 reflections with *I* > 2σ(*I*)
*R*_int_ = 0.044

#### Refinement

*R*[*F* ^2^ > 2σ(*F* ^2^)] = 0.043
*wR*(*F* ^2^) = 0.074
*S* = 1.10
5170 reflections
345 parameters
H-atom parameters constrained
Δρ_max_ = 0.66 e Å^−3^
Δρ_min_ = −0.65 e Å^−3^

### 

Data collection: *CrystalClear* (Rigaku/MSC, 2006 ▶); cell refinement: *CrystalClear*; data reduction: *CrystalClear*; program(s) used to solve structure: *SHELXS97* (Sheldrick, 2008 ▶); program(s) used to refine structure: *SHELXS97* (Sheldrick, 2008 ▶); molecular graphics: *SHELXTL* (Sheldrick, 2008 ▶); software used to prepare material for publication: *SHELXTL*.

## Supplementary Material

Crystal structure: contains datablock(s) global, I. DOI: 10.1107/S160053681102040X/kp2329sup1.cif

Structure factors: contains datablock(s) I. DOI: 10.1107/S160053681102040X/kp2329Isup2.hkl

Additional supplementary materials:  crystallographic information; 3D view; checkCIF report

## Figures and Tables

**Table 1:** Selected bond lengths (Å)

Hg1—Cl1	2.3574 (15)
Hg1—Cl2	2.3687 (19)
Hg1—N3^i^	2.385 (4)
Hg1—N1	2.400 (4)

Symmetry code: (i) 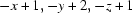.

**Table 2:** Hydrogen-bond geometry (Å, °)

*D*—H⋯*A*	*D*—H	H⋯*A*	*D*⋯*A*	*D*—H⋯*A*
O4—H4⋯N6^ii^	0.82	1.94	2.740 (6)	167
N5—H5*A*⋯O1^iv^	0.86	2.42	3.141 (6)	142

Symmetry codes: (ii) 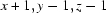; (iii) 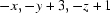; (iv) 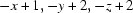.

**Fig. 1.**
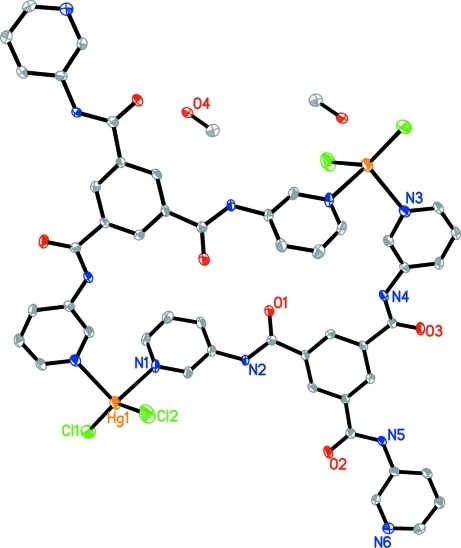
View of the title complex showing the labeling of the non-H atoms. H atoms have been omitted. Symmetry code used to generate the complete molecule: 1-x, 2-y, 1-z.

**Fig. 2.**
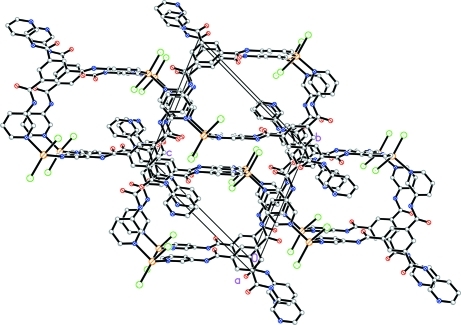
View of the crystal packing along the a axis. Hydrogen bonds are shown as dashed lines.

## supplementary crystallographic information

### Comment

In recent years the rapid progress in supramolecular chemistry has contributed to discovery of  special novel structures being of significance for functional materials (Fortner *et al.* 2005). To control the topology of molecular assemblies, tripodal ligands are proved promising and useful in this area. For example, *N*,*N*',*N*'',-tris(3-pyridinyl)-1,3,5-benzenetricarboxamide (*Z* (Qin *et al.* 2003) has been selected as an excellent tripodal ligand and many intriguing complexes have been successfully accomplished with this ligand. In this work, we selected this ligand as linker, generating a new coordination complex, [Hg~2~(C~24~H~18Ñ~6Õ~3~)~2~Cl~4~]2(CH~3ÕH), (I), which is reported here. In the compound, Hg^II^ atom is four-coordinated by two N atoms from two ligands and two Cl atoms in a distorted tetrahedral coordination sphere (Fig. 1, Table 1). The two Hg^II^ atoms are bridged with two ligands to form a microporous MOFs with 28-number ring. The Hg(II)—N distances are 2.385 (4) Å and 2.400 (4) Å, respectively. The Hg···Hg distance in the ring is 13.568 (5) Å. In the crystal structure, intermolecular hydrogen bonds N2—H—Cl1,  N5—H—O1, and the O4—H—N6 (arising from the CH3OH and ligand) generate the three-dimensional network (Fig. 2, Table 2).

### Experimental

The ligand *N*,*N*',*N*''-tris(3-pyridinyl)-1,3,5-benzenetricarboxamide (0.1 mmol, 0.044 g) in DMF (1 mL) was added dropwise to a solution of HgCl_2_ (0.05 mmol, 0.014 g) in methanol (5 mL). The precipitate was filtered and the resulting solution was allowed to stand at room temperature in the dark. After one week good quality colourless crystals were obtained, separated from a filtrate and dried in air.

### Refinement

H atoms were generated geometrically, with C-H = 0.96,  0.86 and 0.93Å for methyl, N and aromatic H, respectively, and constrained to ride their parent atoms with Uiso(H) = x times Ueq(C), where x = 1.5 for methyl H and x = 1.2 for all other H atoms.

### Crystal data

**Table:**

[Hg_2_Cl_4_(C_24_H_18_N_6_O_3_)_2_]·2CH_4_O	*Z* = 1
*M**_r_* = 1483.95	*F*(000) = 720
Triclinic, *P*1	*D*_x_ = 1.874 Mg m^−^^3^
Hall symbol: -P 1	Mo *K*α radiation, λ = 0.71073 Å
*a* = 8.6772 (17) Å	Cell parameters from 3621 reflections
*b* = 12.243 (2) Å	θ = 2.8–26.0°
*c* = 13.530 (3) Å	µ = 6.10 mm^−^^1^
α = 66.81 (3)°	*T* = 293 K
β = 84.66 (3)°	Prism, colorless
γ = 86.40 (3)°	0.20 × 0.18 × 0.16 mm
*V* = 1315.0 (4) Å^3^	

### Data collection

**Table:**

Rigaku Saturn724 diffractometer	5170 independent reflections
Radiation source: fine-focus sealed tube	4461 reflections with *I* > 2σ(*I*)
graphite	*R*_int_ = 0.044
Detector resolution: 28.5714 pixels mm^-1^	θ_max_ = 26.0°, θ_min_ = 2.8°
dtprofit.ref scans	*h* = −10→10
Absorption correction: numerical (*CrystalClear*; Rigaku/MSC, 2006)	*k* = −15→15
*T*_min_ = 0.738, *T*_max_ = 1.000	*l* = −16→16
14489 measured reflections	

### Refinement

**Table:**

Refinement on *F*^2^	Primary atom site location: structure-invariant direct methods
Least-squares matrix: full	Secondary atom site location: difference Fourier map
*R*[*F*^2^ > 2σ(*F*^2^)] = 0.043	Hydrogen site location: inferred from neighbouring sites
*wR*(*F*^2^) = 0.074	H-atom parameters constrained
*S* = 1.10	*w* = 1/[σ^2^(*F*_o_^2^) + (0.0211*P*)^2^ + 1.2163*P*] where *P* = (*F*_o_^2^ + 2*F*_c_^2^)/3
5170 reflections	(Δ/σ)_max_ = 0.001
345 parameters	Δρ_max_ = 0.66 e Å^−^^3^
0 restraints	Δρ_min_ = −0.65 e Å^−^^3^

### Special details

**Table:**

Geometry. All e.s.d.'s (except the e.s.d. in the dihedral angle between two l.s. planes) are estimated using the full covariance matrix. The cell e.s.d.'s are taken into account individually in the estimation of e.s.d.'s in distances, angles and torsion angles; correlations between e.s.d.'s in cell parameters are only used when they are defined by crystal symmetry. An approximate (isotropic) treatment of cell e.s.d.'s is used for estimating e.s.d.'s involving l.s. planes.
Refinement. Refinement of *F*^2^ against ALL reflections. The weighted *R*-factor *wR* and goodness of fit *S* are based on *F*^2^, conventional *R*-factors *R* are based on *F*, with *F* set to zero for negative *F*^2^. The threshold expression of *F*^2^ > σ(*F*^2^) is used only for calculating *R*-factors(gt) *etc*. and is not relevant to the choice of reflections for refinement. *R*-factors based on *F*^2^ are statistically about twice as large as those based on *F*, and *R*- factors based on ALL data will be even larger.

### Fractional atomic coordinates and isotropic or equivalent isotropic displacement parameters (Å^2^)

**Table:**

	*x*	*y*	*z*	*U*_iso_*/*U*_eq_	
Hg1	0.06417 (3)	1.50692 (2)	0.291708 (18)	0.04562 (9)	
Cl1	0.01030 (19)	1.70597 (13)	0.26971 (13)	0.0578 (4)	
Cl2	−0.0589 (2)	1.32182 (15)	0.35590 (16)	0.0730 (5)	
O1	0.6022 (4)	1.1328 (4)	0.6921 (3)	0.0588 (12)	
O2	0.0476 (5)	1.1393 (4)	1.0191 (3)	0.0609 (13)	
O3	0.5110 (4)	0.6889 (3)	1.1175 (3)	0.0447 (10)	
O4	0.5046 (4)	0.2218 (4)	0.2685 (3)	0.0502 (11)	
H4	0.5939	0.1950	0.2746	0.075*	
N1	0.2878 (5)	1.4403 (4)	0.3940 (3)	0.0388 (11)	
N2	0.3666 (5)	1.2240 (4)	0.6615 (3)	0.0328 (10)	
H2A	0.2743	1.2254	0.6904	0.039*	
N3	0.8189 (5)	0.4833 (4)	0.8782 (3)	0.0396 (11)	
N4	0.5735 (5)	0.6980 (4)	0.9476 (3)	0.0370 (11)	
H4A	0.5560	0.7394	0.8815	0.044*	
N5	0.1166 (5)	0.9886 (4)	1.1696 (3)	0.0383 (11)	
H5A	0.1840	0.9316	1.1933	0.046*	
N6	−0.2183 (5)	1.0971 (4)	1.2982 (4)	0.0440 (12)	
C1	0.4282 (7)	1.4747 (5)	0.3503 (4)	0.0470 (15)	
H1	0.4392	1.5331	0.2809	0.056*	
C2	0.5569 (7)	1.4272 (5)	0.4037 (5)	0.0519 (16)	
H2	0.6543	1.4513	0.3699	0.062*	
C3	0.5436 (6)	1.3435 (5)	0.5077 (4)	0.0431 (14)	
H3	0.6310	1.3113	0.5454	0.052*	
C4	0.3975 (6)	1.3086 (4)	0.5546 (4)	0.0324 (12)	
C5	0.2711 (6)	1.3585 (5)	0.4947 (4)	0.0356 (13)	
H5	0.1722	1.3343	0.5253	0.043*	
C6	0.4667 (6)	1.1419 (4)	0.7223 (4)	0.0321 (12)	
C7	0.4036 (5)	1.0577 (4)	0.8305 (4)	0.0267 (11)	
C8	0.2881 (5)	1.0880 (4)	0.8931 (4)	0.0282 (11)	
H8	0.2388	1.1625	0.8663	0.034*	
C9	0.2452 (5)	1.0064 (4)	0.9972 (4)	0.0269 (11)	
C10	0.3142 (5)	0.8937 (4)	1.0355 (4)	0.0283 (11)	
H10	0.2839	0.8393	1.1042	0.034*	
C11	0.4282 (5)	0.8615 (4)	0.9720 (4)	0.0267 (11)	
C12	0.4739 (5)	0.9443 (4)	0.8704 (4)	0.0299 (12)	
H12	0.5522	0.9241	0.8284	0.036*	
C13	0.1274 (6)	1.0525 (5)	1.0623 (4)	0.0329 (12)	
C14	0.0080 (5)	1.0042 (4)	1.2477 (4)	0.0298 (12)	
C15	−0.1147 (6)	1.0857 (4)	1.2230 (4)	0.0342 (12)	
H15	−0.1257	1.1345	1.1511	0.041*	
C16	−0.1994 (7)	1.0275 (5)	1.3999 (5)	0.0476 (15)	
H16	−0.2682	1.0372	1.4530	0.057*	
C17	−0.0839 (6)	0.9419 (5)	1.4314 (4)	0.0427 (14)	
H17	−0.0782	0.8923	1.5036	0.051*	
C18	0.0221 (6)	0.9311 (5)	1.3551 (4)	0.0366 (13)	
H18	0.1032	0.8752	1.3747	0.044*	
C19	0.5064 (6)	0.7406 (4)	1.0210 (4)	0.0291 (11)	
C20	0.6681 (6)	0.5949 (4)	0.9664 (4)	0.0325 (12)	
C21	0.7074 (6)	0.5125 (5)	1.0650 (4)	0.0408 (14)	
H21	0.6710	0.5214	1.1284	0.049*	
C22	0.8021 (7)	0.4163 (5)	1.0679 (4)	0.0463 (15)	
H22	0.8300	0.3597	1.1337	0.056*	
C23	0.8552 (6)	0.4041 (5)	0.9739 (4)	0.0416 (14)	
H23	0.9182	0.3386	0.9773	0.050*	
C24	0.7266 (6)	0.5763 (5)	0.8752 (4)	0.0429 (15)	
H24	0.7003	0.6314	0.8083	0.051*	
C25	0.4092 (7)	0.1503 (6)	0.3571 (5)	0.0653 (19)	
H25A	0.4394	0.1553	0.4217	0.098*	
H25B	0.4196	0.0693	0.3635	0.098*	
H25C	0.3034	0.1774	0.3469	0.098*	

### Atomic displacement parameters (Å^2^)

**Table:**

	*U*^11^	*U*^22^	*U*^33^	*U*^12^	*U*^13^	*U*^23^
Hg1	0.05767 (17)	0.03596 (14)	0.03701 (14)	0.01382 (10)	−0.00540 (11)	−0.00942 (10)
Cl1	0.0633 (10)	0.0361 (8)	0.0662 (11)	0.0097 (7)	0.0067 (8)	−0.0158 (8)
Cl2	0.0673 (11)	0.0461 (10)	0.0887 (14)	−0.0063 (9)	−0.0155 (10)	−0.0055 (9)
O1	0.040 (2)	0.053 (3)	0.044 (3)	0.016 (2)	0.011 (2)	0.017 (2)
O2	0.078 (3)	0.046 (3)	0.035 (2)	0.040 (2)	0.006 (2)	0.003 (2)
O3	0.066 (3)	0.031 (2)	0.024 (2)	0.0152 (19)	0.0028 (19)	−0.0008 (17)
O4	0.048 (2)	0.056 (3)	0.038 (2)	0.023 (2)	−0.003 (2)	−0.012 (2)
N1	0.049 (3)	0.034 (3)	0.021 (2)	0.003 (2)	−0.005 (2)	0.001 (2)
N2	0.028 (2)	0.034 (2)	0.021 (2)	0.0056 (19)	−0.0017 (18)	0.0043 (19)
N3	0.046 (3)	0.037 (3)	0.032 (3)	0.016 (2)	−0.004 (2)	−0.012 (2)
N4	0.043 (3)	0.032 (2)	0.026 (2)	0.018 (2)	−0.005 (2)	−0.003 (2)
N5	0.045 (3)	0.030 (2)	0.027 (2)	0.020 (2)	0.005 (2)	−0.002 (2)
N6	0.041 (3)	0.047 (3)	0.038 (3)	0.008 (2)	0.001 (2)	−0.012 (2)
C1	0.056 (4)	0.037 (3)	0.028 (3)	0.003 (3)	0.011 (3)	0.005 (3)
C2	0.043 (4)	0.051 (4)	0.042 (4)	−0.003 (3)	0.009 (3)	0.000 (3)
C3	0.036 (3)	0.040 (3)	0.037 (3)	0.004 (3)	−0.005 (3)	0.002 (3)
C4	0.038 (3)	0.027 (3)	0.025 (3)	0.001 (2)	−0.001 (2)	−0.002 (2)
C5	0.035 (3)	0.036 (3)	0.026 (3)	−0.004 (2)	0.001 (2)	−0.002 (2)
C6	0.029 (3)	0.028 (3)	0.031 (3)	0.000 (2)	0.002 (2)	−0.004 (2)
C7	0.028 (3)	0.023 (3)	0.023 (3)	0.002 (2)	−0.004 (2)	−0.002 (2)
C8	0.027 (3)	0.023 (3)	0.031 (3)	0.006 (2)	−0.005 (2)	−0.006 (2)
C9	0.031 (3)	0.026 (3)	0.021 (3)	0.005 (2)	−0.003 (2)	−0.007 (2)
C10	0.030 (3)	0.025 (3)	0.025 (3)	−0.001 (2)	−0.001 (2)	−0.005 (2)
C11	0.026 (3)	0.025 (3)	0.026 (3)	0.008 (2)	−0.006 (2)	−0.007 (2)
C12	0.028 (3)	0.029 (3)	0.028 (3)	0.000 (2)	0.004 (2)	−0.007 (2)
C13	0.034 (3)	0.028 (3)	0.032 (3)	0.004 (2)	−0.001 (2)	−0.009 (2)
C14	0.026 (3)	0.029 (3)	0.035 (3)	0.003 (2)	0.000 (2)	−0.014 (2)
C15	0.037 (3)	0.031 (3)	0.026 (3)	0.003 (2)	0.003 (2)	−0.003 (2)
C16	0.045 (4)	0.057 (4)	0.036 (3)	0.009 (3)	0.008 (3)	−0.017 (3)
C17	0.050 (4)	0.046 (4)	0.028 (3)	0.009 (3)	−0.003 (3)	−0.012 (3)
C18	0.037 (3)	0.032 (3)	0.035 (3)	0.010 (2)	−0.006 (3)	−0.008 (3)
C19	0.031 (3)	0.024 (3)	0.028 (3)	0.002 (2)	0.002 (2)	−0.007 (2)
C20	0.034 (3)	0.025 (3)	0.034 (3)	0.007 (2)	−0.001 (2)	−0.008 (2)
C21	0.053 (4)	0.036 (3)	0.022 (3)	0.009 (3)	0.001 (3)	−0.002 (2)
C22	0.058 (4)	0.031 (3)	0.033 (3)	0.013 (3)	0.000 (3)	0.002 (3)
C23	0.047 (3)	0.029 (3)	0.040 (3)	0.015 (3)	−0.005 (3)	−0.005 (3)
C24	0.051 (4)	0.040 (3)	0.029 (3)	0.021 (3)	−0.004 (3)	−0.007 (3)
C25	0.060 (4)	0.072 (5)	0.053 (4)	0.014 (4)	0.004 (4)	−0.016 (4)

### Geometric parameters (Å, °)

**Table:**

Hg1—Cl1	2.3574 (15)	C5—H5	0.9300
Hg1—Cl2	2.3687 (19)	C6—C7	1.496 (6)
Hg1—N3^i^	2.385 (4)	C7—C8	1.380 (6)
Hg1—N1	2.400 (4)	C7—C12	1.399 (6)
O1—C6	1.221 (6)	C8—C9	1.401 (6)
O2—C13	1.203 (6)	C8—H8	0.9300
O3—C19	1.209 (6)	C9—C10	1.386 (6)
O4—C25	1.404 (7)	C9—C13	1.513 (7)
O4—H4	0.8200	C10—C11	1.390 (6)
N1—C1	1.321 (7)	C10—H10	0.9300
N1—C5	1.336 (6)	C11—C12	1.388 (6)
N2—C6	1.347 (6)	C11—C19	1.509 (6)
N2—C4	1.421 (6)	C12—H12	0.9300
N2—H2A	0.8600	C14—C15	1.382 (7)
N3—C23	1.329 (6)	C14—C18	1.387 (7)
N3—C24	1.340 (6)	C15—H15	0.9300
N3—Hg1^i^	2.385 (4)	C16—C17	1.371 (7)
N4—C19	1.363 (6)	C16—H16	0.9300
N4—C20	1.410 (6)	C17—C18	1.358 (7)
N4—H4A	0.8600	C17—H17	0.9300
N5—C13	1.348 (6)	C18—H18	0.9300
N5—C14	1.411 (6)	C20—C21	1.377 (7)
N5—H5A	0.8600	C20—C24	1.386 (7)
N6—C16	1.322 (7)	C21—C22	1.383 (7)
N6—C15	1.338 (6)	C21—H21	0.9300
C1—C2	1.359 (8)	C22—C23	1.372 (7)
C1—H1	0.9300	C22—H22	0.9300
C2—C3	1.375 (7)	C23—H23	0.9300
C2—H2	0.9300	C24—H24	0.9300
C3—C4	1.377 (7)	C25—H25A	0.9600
C3—H3	0.9300	C25—H25B	0.9600
C4—C5	1.387 (7)	C25—H25C	0.9600
			
Cl1—Hg1—Cl2	140.17 (6)	C9—C10—H10	119.8
Cl1—Hg1—N3^i^	105.41 (12)	C11—C10—H10	119.8
Cl2—Hg1—N3^i^	101.24 (13)	C12—C11—C10	119.1 (4)
Cl1—Hg1—N1	107.24 (12)	C12—C11—C19	122.8 (4)
Cl2—Hg1—N1	97.44 (12)	C10—C11—C19	117.9 (4)
N3^i^—Hg1—N1	98.31 (15)	C11—C12—C7	120.8 (4)
C25—O4—H4	109.5	C11—C12—H12	119.6
C1—N1—C5	119.3 (5)	C7—C12—H12	119.6
C1—N1—Hg1	121.6 (4)	O2—C13—N5	123.2 (5)
C5—N1—Hg1	118.9 (4)	O2—C13—C9	121.0 (5)
C6—N2—C4	126.8 (4)	N5—C13—C9	115.7 (4)
C6—N2—H2A	116.6	C15—C14—C18	118.0 (4)
C4—N2—H2A	116.6	C15—C14—N5	123.7 (5)
C23—N3—C24	118.1 (4)	C18—C14—N5	118.2 (4)
C23—N3—Hg1^i^	125.5 (3)	N6—C15—C14	122.7 (5)
C24—N3—Hg1^i^	115.6 (3)	N6—C15—H15	118.6
C19—N4—C20	128.3 (4)	C14—C15—H15	118.6
C19—N4—H4A	115.9	N6—C16—C17	123.6 (5)
C20—N4—H4A	115.9	N6—C16—H16	118.2
C13—N5—C14	127.8 (4)	C17—C16—H16	118.2
C13—N5—H5A	116.1	C18—C17—C16	118.8 (5)
C14—N5—H5A	116.1	C18—C17—H17	120.6
C16—N6—C15	117.5 (5)	C16—C17—H17	120.6
N1—C1—C2	121.9 (5)	C17—C18—C14	119.3 (5)
N1—C1—H1	119.1	C17—C18—H18	120.4
C2—C1—H1	119.1	C14—C18—H18	120.4
C1—C2—C3	120.3 (5)	O3—C19—N4	124.0 (4)
C1—C2—H2	119.9	O3—C19—C11	121.6 (4)
C3—C2—H2	119.9	N4—C19—C11	114.3 (4)
C2—C3—C4	118.2 (5)	C21—C20—C24	117.7 (5)
C2—C3—H3	120.9	C21—C20—N4	126.6 (5)
C4—C3—H3	120.9	C24—C20—N4	115.7 (4)
C3—C4—C5	118.7 (5)	C20—C21—C22	118.6 (5)
C3—C4—N2	124.2 (5)	C20—C21—H21	120.7
C5—C4—N2	117.1 (4)	C22—C21—H21	120.7
N1—C5—C4	121.7 (5)	C23—C22—C21	120.2 (5)
N1—C5—H5	119.1	C23—C22—H22	119.9
C4—C5—H5	119.1	C21—C22—H22	119.9
O1—C6—N2	123.2 (5)	N3—C23—C22	121.8 (5)
O1—C6—C7	120.1 (4)	N3—C23—H23	119.1
N2—C6—C7	116.7 (4)	C22—C23—H23	119.1
C8—C7—C12	119.6 (4)	N3—C24—C20	123.6 (5)
C8—C7—C6	123.8 (4)	N3—C24—H24	118.2
C12—C7—C6	116.5 (4)	C20—C24—H24	118.2
C7—C8—C9	119.9 (4)	O4—C25—H25A	109.5
C7—C8—H8	120.1	O4—C25—H25B	109.5
C9—C8—H8	120.1	H25A—C25—H25B	109.5
C10—C9—C8	120.0 (4)	O4—C25—H25C	109.5
C10—C9—C13	124.5 (4)	H25A—C25—H25C	109.5
C8—C9—C13	115.4 (4)	H25B—C25—H25C	109.5
C9—C10—C11	120.5 (4)		
			
Cl1—Hg1—N1—C1	−74.8 (4)	C6—C7—C12—C11	177.1 (4)
Cl2—Hg1—N1—C1	136.9 (4)	C14—N5—C13—O2	5.0 (9)
N3^i^—Hg1—N1—C1	34.3 (4)	C14—N5—C13—C9	−173.5 (5)
Cl1—Hg1—N1—C5	111.1 (4)	C10—C9—C13—O2	−163.7 (5)
Cl2—Hg1—N1—C5	−37.2 (4)	C8—C9—C13—O2	18.9 (8)
N3^i^—Hg1—N1—C5	−139.8 (4)	C10—C9—C13—N5	14.9 (8)
C5—N1—C1—C2	1.7 (9)	C8—C9—C13—N5	−162.6 (5)
Hg1—N1—C1—C2	−172.4 (5)	C13—N5—C14—C15	4.9 (9)
N1—C1—C2—C3	−2.2 (10)	C13—N5—C14—C18	−177.3 (5)
C1—C2—C3—C4	1.0 (9)	C16—N6—C15—C14	0.7 (8)
C2—C3—C4—C5	0.6 (8)	C18—C14—C15—N6	0.7 (8)
C2—C3—C4—N2	−179.4 (5)	N5—C14—C15—N6	178.5 (5)
C6—N2—C4—C3	−20.0 (8)	C15—N6—C16—C17	−2.7 (9)
C6—N2—C4—C5	160.0 (5)	N6—C16—C17—C18	3.3 (10)
C1—N1—C5—C4	0.0 (8)	C16—C17—C18—C14	−1.8 (9)
Hg1—N1—C5—C4	174.2 (4)	C15—C14—C18—C17	0.0 (8)
C3—C4—C5—N1	−1.1 (8)	N5—C14—C18—C17	−178.0 (5)
N2—C4—C5—N1	178.9 (5)	C20—N4—C19—O3	−5.2 (9)
C4—N2—C6—O1	2.2 (9)	C20—N4—C19—C11	172.9 (5)
C4—N2—C6—C7	−176.4 (5)	C12—C11—C19—O3	150.9 (5)
O1—C6—C7—C8	148.0 (5)	C10—C11—C19—O3	−23.8 (7)
N2—C6—C7—C8	−33.4 (7)	C12—C11—C19—N4	−27.2 (7)
O1—C6—C7—C12	−28.5 (7)	C10—C11—C19—N4	158.1 (4)
N2—C6—C7—C12	150.0 (5)	C19—N4—C20—C21	2.8 (9)
C12—C7—C8—C9	1.8 (7)	C19—N4—C20—C24	−177.1 (5)
C6—C7—C8—C9	−174.6 (5)	C24—C20—C21—C22	−0.1 (8)
C7—C8—C9—C10	−2.7 (7)	N4—C20—C21—C22	−180.0 (5)
C7—C8—C9—C13	174.9 (4)	C20—C21—C22—C23	0.0 (9)
C8—C9—C10—C11	1.2 (7)	C24—N3—C23—C22	−0.7 (9)
C13—C9—C10—C11	−176.1 (5)	Hg1^i^—N3—C23—C22	168.6 (4)
C9—C10—C11—C12	1.0 (7)	C21—C22—C23—N3	0.4 (9)
C9—C10—C11—C19	176.0 (4)	C23—N3—C24—C20	0.6 (9)
C10—C11—C12—C7	−1.9 (7)	Hg1^i^—N3—C24—C20	−169.7 (4)
C19—C11—C12—C7	−176.5 (5)	C21—C20—C24—N3	−0.2 (9)
C8—C7—C12—C11	0.4 (8)	N4—C20—C24—N3	179.7 (5)

Symmetry codes: (i) −*x*+1, −*y*+2, −*z*+1.

### Hydrogen-bond geometry (Å, °)

**Table:**

*D*—H···*A*	*D*—H	H···*A*	*D*···*A*	*D*—H···*A*
O4—H4···N6^ii^	0.82	1.94	2.740 (6)	167.
N2—H2A···Cl1^iii^	0.86	2.64	3.465 (4)	162.
N5—H5A···O1^iv^	0.86	2.42	3.141 (6)	142.

Symmetry codes: (ii) *x*+1, *y*−1, *z*−1; (iii) −*x*, −*y*+3, −*z*+1; (iv) −*x*+1, −*y*+2, −*z*+2.
